# MAPK8 and CAPN1 as potential biomarkers of intervertebral disc degeneration overlapping immune infiltration, autophagy, and ceRNA

**DOI:** 10.3389/fimmu.2023.1188774

**Published:** 2023-05-30

**Authors:** Yuxin Zhang, Jiahui Zhang, Zhongyi Sun, Hui Wang, Ruonan Ning, Longyu Xu, Yichen Zhao, Kai Yang, Xiaobing Xi, Jiwei Tian

**Affiliations:** ^1^ School of Medicine, Shanghai University, Shanghai, China; ^2^ Department of Orthopaedics, Shanghai Key Laboratory for Prevention and Treatment of Bone and Joint Diseases, Shanghai Institute of Traumatology and Orthopaedics, Ruijin Hospital, Shanghai Jiao Tong University School of Medicine, Shanghai, China; ^3^ Department of Orthopedics, BenQ Medical Center, The Affiliated BenQ Hospital of Nanjing Medical University, Nanjing, China; ^4^ Department of Orthopaedics, Shanghai Changzheng Hospital, Shanghai, China; ^5^ Department of Neurosurgery, Ruijin Hospital, Shanghai Jiaotong University School of Medicine, Shanghai, China

**Keywords:** intervertebral disc degeneration (IDD), immune infiltration, bioinformatic analysis, autophagy, MAPK8, CAPN1

## Abstract

**Background:**

Intervertebral disc degeneration (IDD) is one of the most common health problems in the elderly and a major causative factor in low back pain (LBP). An increasing number of studies have shown that IDD is closely associated with autophagy and immune dysregulation. Therefore, the aim of this study was to identify autophagy-related biomarkers and gene regulatory networks in IDD and potential therapeutic targets.

**Methods:**

We obtained the gene expression profiles of IDD by downloading the datasets GSE176205 and GSE167931 from the Gene Expression Omnibus (GEO) public database. Subsequently, differentially expressed genes (DEGs) analysis, Kyoto Encyclopedia of Genes and Genomes (KEGG) analysis, gene ontology (GO), and gene set enrichment analysis (GSEA) were performed to explore the biological functions of DEGs. Differentially expressed autophagy-related genes (DE-ARGs) were then crossed with the autophagy gene database. The hub genes were screened using the DE-ARGs protein–protein interaction (PPI) network. The correlation between the hub genes and immune infiltration and the construction of the gene regulatory network of the hub genes were confirmed. Finally, quantitative PCR (qPCR) was used to validate the correlation of hub genes in a rat IDD model.

**Results:**

We obtained 636 DEGs enriched in the autophagy pathway. Our analysis revealed 30 DE-ARGs, of which six hub genes (*MAPK8*, *CTSB*, *PRKCD*, *SNCA*, *CAPN1*, and *EGFR*) were identified using the MCODE plugin. Immune cell infiltration analysis revealed that there was an increased proportion of CD8^+^ T cells and M0 macrophages in IDD, whereas CD4^+^ memory T cells, neutrophils, resting dendritic cells, follicular helper T cells, and monocytes were much less abundant. Subsequently, the competitive endogenous RNA (ceRNA) network was constructed using 15 long non-coding RNAs (lncRNAs) and 21 microRNAs (miRNAs). In quantitative PCR (qPCR) validation, two hub genes, *MAPK8* and *CAPN1*, were shown to be consistent with the bioinformatic analysis results.

**Conclusion:**

Our study identified *MAPK8* and *CAPN1* as key biomarkers of IDD. These key hub genes may be potential therapeutic targets for IDD.

## Introduction

Intervertebral disc degeneration (IDD) is a major global public health problem (1) that is frequently observed in middle-aged and elderly people (2) and is the pathological basis of a number of degenerative spinal conditions and diseases, such as low back pain (LBP) (3), disc herniation (4), and cervical spondylosis (5). IDD is a degenerative musculoskeletal disease associated with multiple factors and has a complex and multifaceted pathogenesis that mainly includes excessive mechanical stress (6), excessive apoptosis of nucleus pulposus (NP) cells (7), abnormal degradation of the extracellular matrix (ECM) (8), inflammatory response (9), autophagy disorders (10), oxidative stress injury (11), and genetics (12). The current therapies for symptom management and pain control cannot heal the injured disc or prevent the progression of IDD (13, 14). The primary concern of our study was identifying what molecules are involved and what biological functions are related to the process of IDD, as potential IDD therapeutic targets.

Autophagy is the biological process for the self-degradation and recycling of cellular components; it relies on lysosomes to clear redundant protein polymers and damaged organelles, such as mitochondria and peroxisomes (15–18). Many diseases, notably cancer, diabetes, heart disease, and muscle disease, are related to autophagy disruption (10, 19, 20). An increasing number of studies have shown that there is a close relationship between autophagy and IDD, and it has been reported that autophagy-related apoptosis promotes the progression of IDD (10). In addition, there is a significant correlation between the progression of IDD and immune cell infiltration (21). Moreover, in recent years, particular attention has been given to non-coding RNAs, including microRNAs (miRNAs) and long non-coding RNAs (lncRNAs), which revealed that they play significant roles in the initiation and progression of IDD (22, 23).

Although many studies have provided preliminary evidence for the regulatory role that autophagy dysregulation plays in the development of IDD, most studies have focused only on the biological functions performed by specific genes and have not focused on their integration with the altered immune microenvironment within the intervertebral disc and the regulation of target gene expression by non-coding RNAs. Based on a high-throughput sequencing dataset of IDD patients obtained from public databases, we performed bioinformatics analysis to identify the hub genes associated with immune infiltration and autophagy dysfunction in the development of IDD and performed competing endogenous RNA (ceRNA) network construction for the hub genes. Finally, PCR validation confirmed the relevance of MAPK8 and CAPN1 in the IDD pathogenesis.

## Material and methods

### Data source and processing

**Figure 1:**
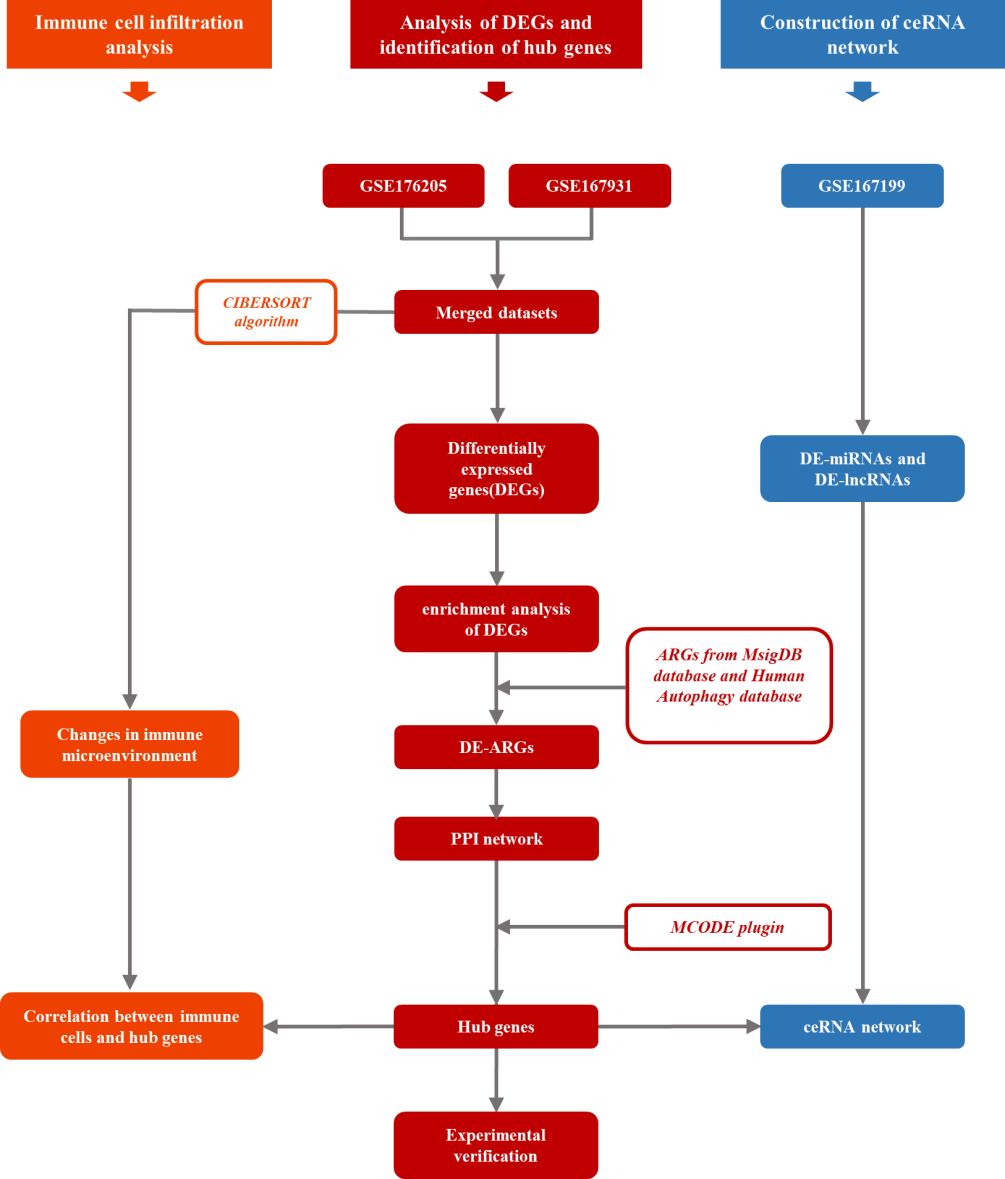
The workflow of this study.

In this study, three IDD-related high-throughput RNA sequencing datasets were obtained from the Gene Expression Omnibus (GEO; https://www.ncbi.nlm.nih.gov/geo/) database, that is, GSE176205, GSE167931, and GSE167199. The detailed contents of three series are listed in **Supplementary Table S1**. Both the GSE176205 (containing three controls and six IDD samples) and GSE167931 (containing four controls and five IDD samples) datasets were applied for differentially expressed genes (DEGs) analysis and normalized using the normalizeBetweenArrays function *via* the limma R package. The two preprocessed datasets mentioned above were combined, and batch effects were removed using the sva R package with the ComBat function. After obtaining the combined dataset, the DEGs of the control and IDD samples were analyzed using the limma R package, with |log2FC| > 1.5 and *p* < 0.05 being used as screening thresholds. The GSE167199 dataset contained three controls and three IDD samples that were applied for microRNAs and lncRNA analysis. The GENCODE database was used for lncRNA annotation. **Figure 1** shows the workflow of this study.

### Functional enrichment analysis of DEGs

The DAVID database (https://david.ncifcrf.gov/) was used to identify biological terms that were enriched with DEGs, filtering out statistically significant gene ontology (GO) terms and Kyoto Encyclopedia of Genes and Genomes (KEGG) pathways at a threshold of *p* < 0.05. All results were plotted using the ggplot2 R package.

### Gene set enrichment analysis

Gene set enrichment analysis (GSEA) of DEGs obtained from the differential analysis was performed to explore the potential biological processes of DEGs involved in IDD. The hallmark gene set was obtained from the MSigDB database, and GSEA analysis was performed using the ClusterProfiler R package (24). The gene sets of the DEGs were sorted according to log2FC, and the normalized enrichment score (NES) in GSEA was obtained. | NES | > 1 and *p* < 0.05 were considered to indicate significantly enriched pathways.

### Identification and functional enrichment of differentially expressed autophagy-related genes

We selected C5 ontology gene sets in the MsigDB database as the filtering criteria, and the 404 genes from the 11 gene sets that are related to autophagy were obtained. In addition, 232 genes involved in autophagy were obtained from the Human Autophagy database (http://www.autophagy.lu/index.html). The obtained gene sets were combined to create a gene set containing 547 ARGs. The intersection of ARGs and DEGs was used to obtain the differentially expressed autophagy-related genes (DE-ARGs). KEGG and GO analyses of DE-ARGs were performed using the DAVID database, with *p* < 0.05 as the threshold.

### Construction of protein–protein interaction network

The Search Tool for the Retrieval of Interacting Genes/proteins (STRING; https://string-db.org/) database contains both the known and predicted protein interactions; these were used to construct the PPI network. The DE-ARGs list was imported into the STRING online platform, and PPI analysis was performed with the default settings and imported into Cytoscape 3.9.1 software. The hub PPI network was screened and visualized using the MCODE analysis plugin. Genes from the hub PPI network were treated as hub genes for subsequent analysis. Receiver operating characteristic (ROC) curve analysis was used to evaluate the diagnostic performance of the hub genes. ROC analysis was carried out using the pROC package in R.

### Immune cell infiltration analysis

The CIBERSORT algorithm, which is used to evaluate changes in immune cells during immune infiltration, was used in this study to investigate changes in the relative proportion of immune cell infiltration in IDD. We performed a correlation analysis of immune cell infiltration and hub gene expression levels using the psych package in R.

### Construction of ceRNA network

We utilized the following procedures to construct the ceRNA network: (1) differential analysis of GSE167199 with the limma package in the R environment using | log2FC | > 1 and p < 0.05 as screening thresholds to obtain differentially expressed microRNAs (DE-miRNAs) and differentially expressed lncRNAs (DE-lncRNAs); (2) using the ENCORI database (https://rna.sysu.edu.cn/encori/index.php) to predict the target miRNAs of the hub gene that intersect with DE-miRNAs to construct the final mRNA–miRNA axis; (3) using mirNet (https://www.mirnet.ca/) to predict the lncRNAs that would be targeted by the DE-miRNAs that bind to the hub gene [the predicted lncRNAs were then taken to intersect with the DE-lncRNAs from (1) to construct the miRNA–lncRNA axis]; and (4) combining the multiple mRNA–miRNA and miRNA–lncRNA axes obtained from the above steps into a mRNA–miRNA–lncRNA regulatory axis according to the predicted binding trends. In addition, the ceRNA network was visualized using Cytoscape software.

### IDD rat models

Twenty-four 8-week-old adult male Sprague Dawley rats were used in this study. The rats were anesthetized by intraperitoneal injection with 2% (w/v) pentobarbital (40 mg/kg) and randomly assigned to either the control group or the IDD group, with an equal number assigned to each group. The caudal discs C7/8 were identified and selected for the study according to the methods used in the previous study (25). A 27-gauge needle punctured discs C7/8, crossing the nucleus pulposus to the contralateral annulus fibrosus. After complete penetration, the needle was rotated twice 360° and held for 60 s. The resistance of the contralateral annulus fibrosus controlled the depth of needle penetration.

### X-rays and MRI

After we anesthetized the rats with isoflurane (3% for induction and 1% for maintenance), we placed them in an induction box in which the air flow rate was adjusted to 1 L/min. Once the rats were fully anesthetized, we removed them from the induction box and placed respiratory masks on them, through which they inhaled isoflurane gas at an adjusted concentration of 1%. The rats were placed in prone positions under the X-ray equipment (Toshiba China, E7252) and in a 7.0 T small-animal MRI system (CG NOVILA 7.0 T, Chenuang, China), and images were collected.

### Hematoxylin and eosin staining

After 4 weeks of feeding, all rats were euthanized, and their caudal intervertebral discs were collected. Half of the samples from each group were fixed in 10% buffered formalin for 48 h, and the other half were frozen and stored at –20°C. Subsequently, the caudal intervertebral disc samples were decalcified in 10% etheylenediaminetetraacetic acid (EDTA) for 14 days and embedded in paraffin. The samples were cut into 4-μm-thick coronal-oriented sections, which were then processed for hematoxylin and eosin (H&E) staining (26, 27).

### Real-time quantitative polymerasechain reaction

Total RNA was extracted from the frozen NP tissue of caudal vertebra using the TRIzol reagent (Invitrogen, CA, USA). PrimeScriptTM RT Master Mix #RR036 A (Takara, Beijing, China) was used to synthesize complementary DNA (cDNA) according to information on the quantity and quality of the RNA. TB Green^®^ Premix Ex Taq™ #RR420B (Takara) was used to perform quantitative PCR (qPCR) on the 7500 Real-Time PCR System (Applied Biosystems, CA, USA). The primer sequences are listed in **Supplementary Table S2**, and the primers were created by Sangon Biotech (Shanghai, China). The relative expression levels of the genes were calculated utilizing the 2^–(ΔΔCT)^ method, with *GAPDH* serving as the internal reference gene for the PCR data.

### Statistical analysis

The data processing, statistical analysis, and graphs were performed or generated *via* R 4.2.1 software and GraphPad Prism 9 software. In all analyses, *p* < 0.05 was regarded as statistically significant.

## Results

### Identification of DEGs

**Figure 2:**
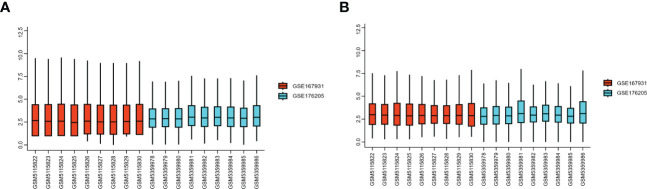
GEO dataset preprocessing. **(A)** Gene expression level of the dataset before preprocessing. **(B)** Gene expression level of the dataset after preprocessing. GEO, Gene Expression Omnibus.

**Figure 3:**
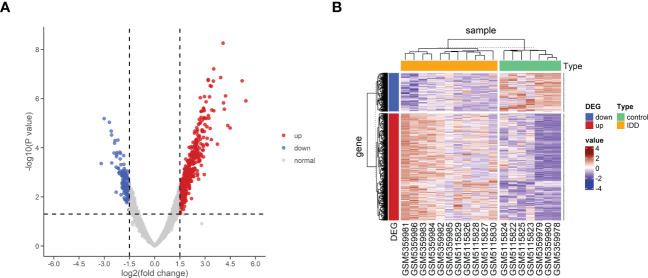
Differential expression of mRNA associated with IDD. **(A)** Volcano map of differentially expressed genes between control and IDD samples. **(B)** Heatmap of differentially expressed genes between control and IDD samples. The scale() function was used to normalize the expressions of the DEGs in R. DEG, differentially expressed genes; IDD, intervertebral disc degeneration; mRNA, messenger RNA.

Two high-throughput sequencing datasets, GSE176205 and GSE167931, were normalized and merged into one dataset for this study. Batch effect elimination was then performed on the merged dataset before data analysis (**Figures 2A, B**). A total of 636 DEGs, containing 468 upregulated genes and 168 downregulated genes, were obtained from the combined dataset using the limma package for differential gene analysis in R (**Figures 3A, B**). All DEGs are shown in **Supplementary Table S3**.

### Enrichment analysis of DEGs

**Figure 4:**
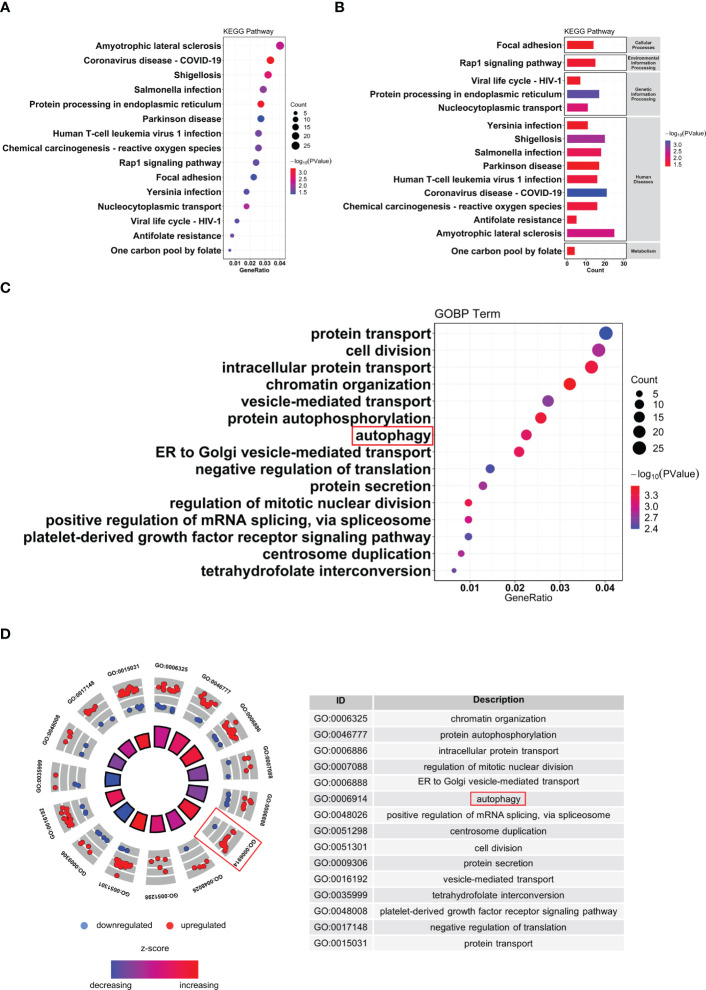
Functional enrichment of analysis of DEGs. **(A, B)** KEGG pathway analysis of DEGs. **(C, D)** GO biological process enrichment results of DEGs. DEG, differentially expressed genes; GO, Gene Ontology; KEGG, Kyoto Encyclopedia of Genes and Genomes.

To further explore the potential biological changes of these screened DEGs, KEGG and GO enrichment analyses were performed using the DAVID online database and exported into the R environment for visualization. The KEGG results showed that the DEGs were mainly enriched in amyotrophic lateral sclerosis, coronavirus disease (COVID-19), shigellosis, *Salmonella* infection, and protein processing in the endoplasmic reticulum. Most of the top 15 KEGG pathways were in immune-related diseases (**Figures 4A, B**). The results of GO BP (biological process) annotation showed that the DEGs were mainly enriched in chromatin organization, protein autophosphorylation, and autophagy. The top 15 GO biological processes revealed that the DEGs were closely associated with autophagy (**Figures 4C, D**).

**Figure 5:**
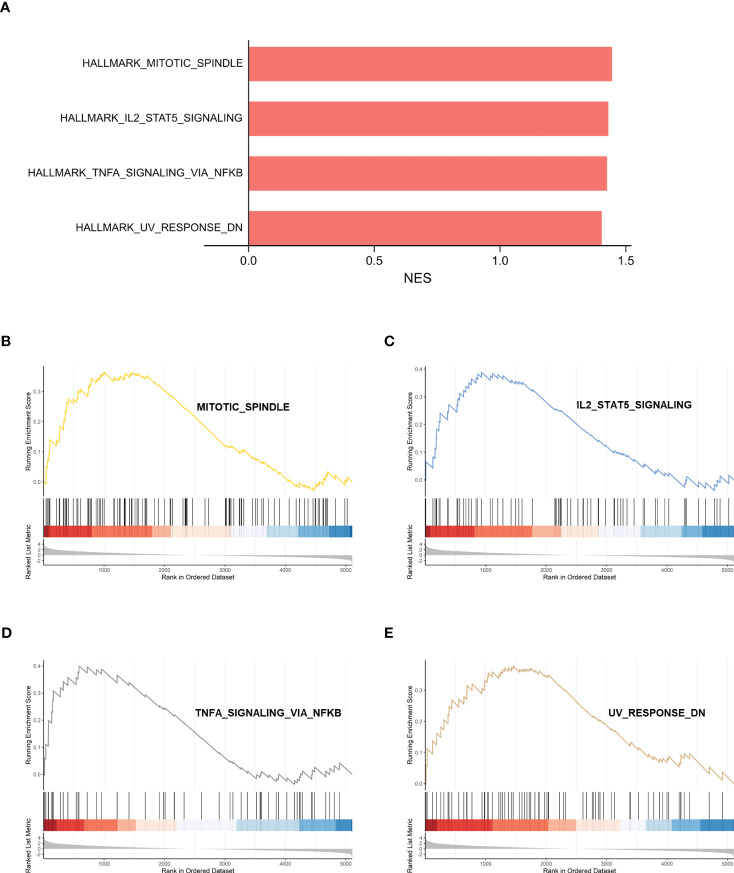
GSEA analysis of HALLMARK between control samples and IDD samples. **(A)** Normalized enrichment scores (NES) for HALLMARK gene sets representing the combined dataset. **(B–E)** Results of GSEA analysis after using threshold screening. GSEA, gene set enrichment analysis; IDD, intervertebral disc degeneration.

The gene set h.all.v2022.1.Hs.symbols was used for GSEA analysis. After excluding the results without statistical significance, MITOTIC_SPINDLE, IL2_STAT5_ SIGNALING, TNFA_SIGNALING_VIA_NFKB, and UV_RESPONSE_DN were significantly activated in IDD (**Figure 5**), suggesting that these biological processes may be closely related to IDD.

### Identification of DE-ARGs

**Figure 6:**
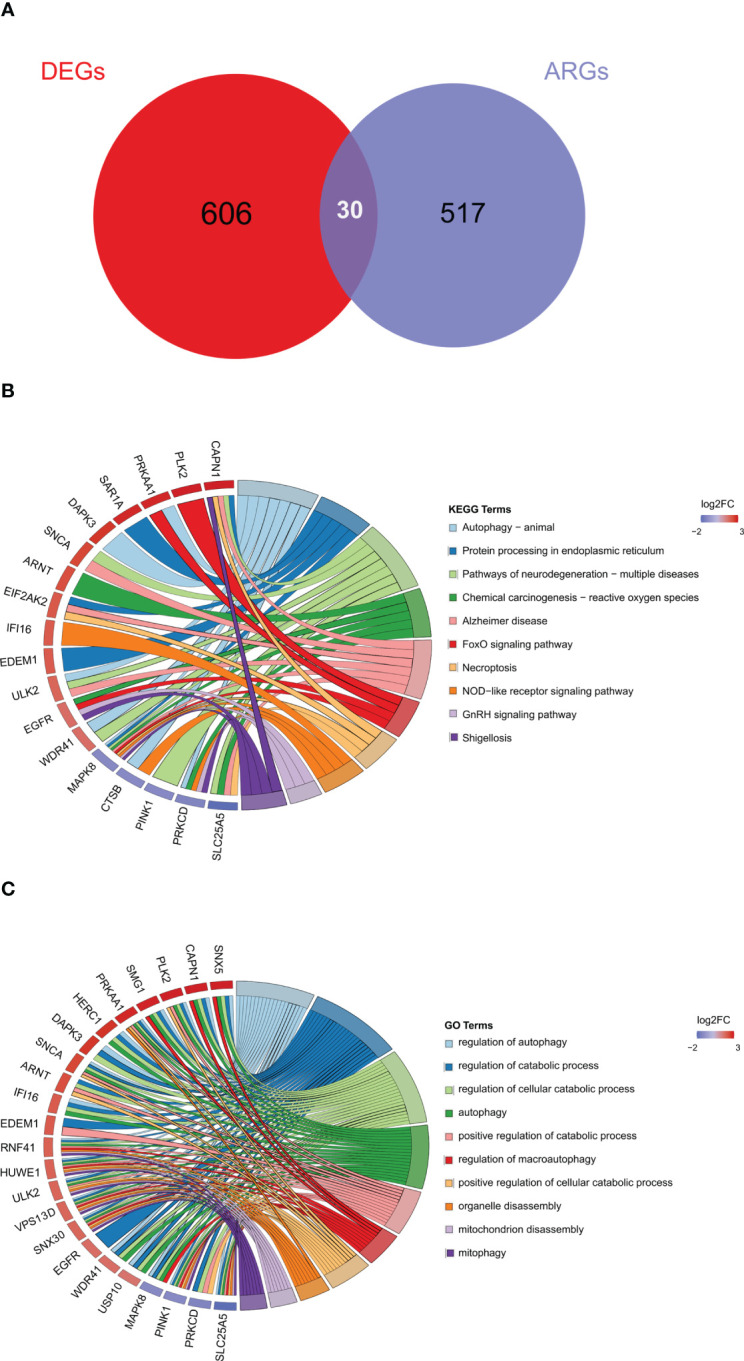
Identification of autophagy-related differentially expressed genes. **(A)** Venn diagram of DE-ARGs between DEGs and ARGs. **(B, C)** KEGG and GO biological process enrichment results of DE-ARGs. ARGS, autophagy-related genes; DE-ARGs, differentially expressed autophagy-related genes; DEGs, differentially expressed genes; GO, Gene Ontology; KEGG, Kyoto Encyclopedia of Genes and Genomes.

To further explore autophagy-related genes (ARGs) in IDD, we combined the 404 ARGs obtained from the MsigDB database with the 232 ARGs obtained from the Human Autophagy database to obtain a total of 547 ARGs. The ARGs were then intersected with the DEGs to obtain the 30 DE-ARGs (**Figure 6A**). KEGG and GO analyses of the DE-ARGs were performed using the DAVID database with *p* < 0.05 as the threshold, and the enrichment results were visualized in R software (**Figures 6B, C**).

### Construction of PPI and identification of hub genes

**Figure 7:**
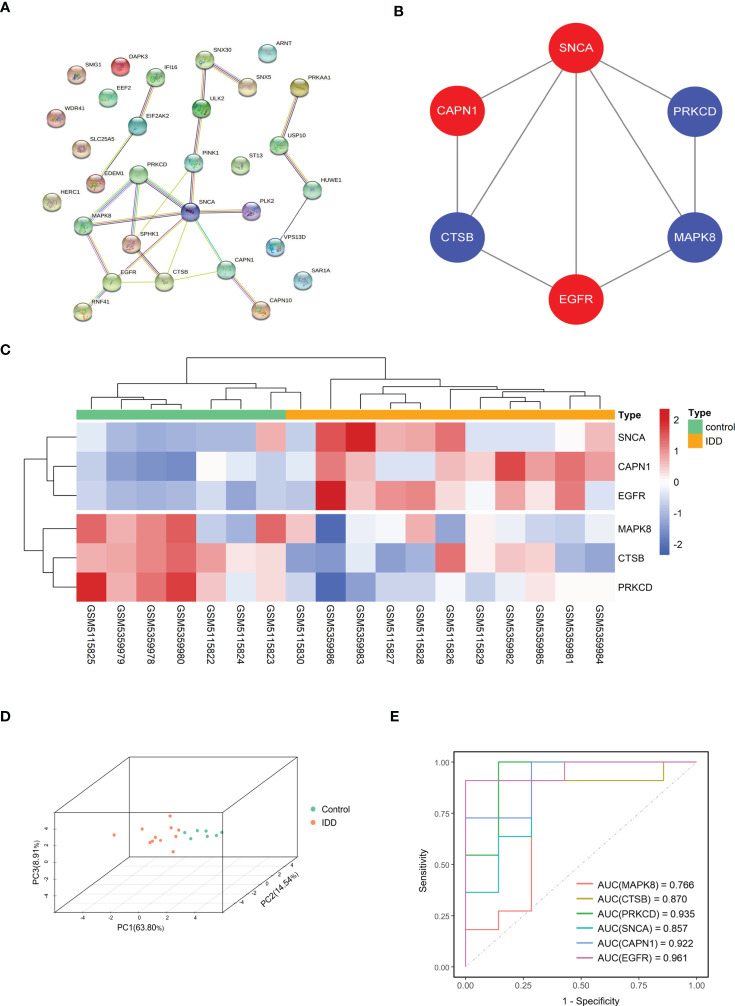
Screening of hub gene in DE-ARGs. **(A)** The protein–protein interaction network visualized by STRING. **(B)** Six hub genes were screened by the MCODE plug-in. Red nodes represent upregulated expressed genes, blue nodes represent downregulated expressed genes. **(C, D)** Gene expression heat map and 3D PCA map of the six hub genes. **(E)** ROC curve of six hub genes in GSE176205 and GSE167931. DE-ARGs, differentially expressed autophagy-related genes; PCA, principal component analysis; ROC, receiver operating characteristic; STRING, Search Tool for the Retrieval of Interacting Genes/Proteins.

We explored the interactions among the 30 DE-ARGs using the STRING database with medium confidence and obtained a PPI network containing 30 nodes and 24 edges (**Figure 7A**). Using Cytoscape’s internal analysis plug-in, MCODE, the PPI network of the DE-ARGs was filtered to gain the subnetworks with the highest clustering scores for visualization (**Figure 7B**). As shown in the figure, we hypothesized that *MAPK8*, *CTSB*, *PRKCD*, *SNCA*, *CAPN1*, and *EGFR* are hub genes.

The heat map and 3D PCA map of these six hub genes in the combined dataset revealed that the control group was significantly different from the IDD group (**Figures 7C, D**). In both the GSE176205 and GSE167931 datasets, the AUC values of the six hub genes were above 0.75, indicating that the six hub genes have excellent diagnostic performance and can be used as promising biomarkers in the diagnosis of IDD (**Figure 7E**).

### The changes in immune cell infiltration in IDD

**Figure 8:**
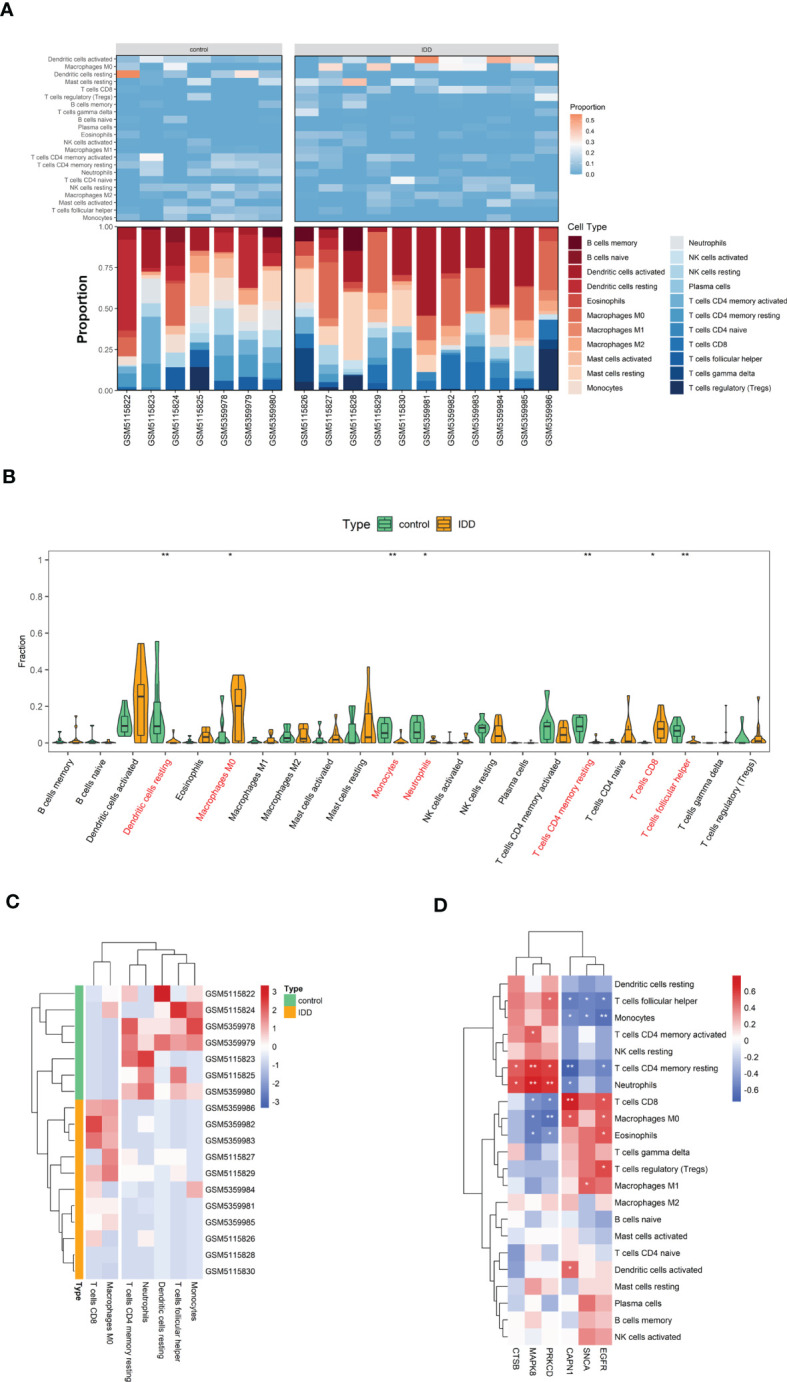
The visualization of cell infiltration between the IDD and control groups. **(A)** The stacked bar chart and heat map indicates the relative proportions of 22 immune cells. **(B)** A violin plot of the immune cell proportions in two groups. **(C)** Significantly different immune cell infiltration in the two sample groups. **(D)** Heat map of the correlation between the expression of the six hub genes and the infiltration of 22 immune cell type proportions. (* p < 0.05, ** p < 0.01) .

We evaluated the relative abundance of infiltrating immune cell subtypes in normal and IDD samples using the CIBERSORT algorithm. The bar graph and heat map show the infiltration of multiple immune cell subtypes in each sample (**Figure 8A**). The violin plot shows the difference in the percentage of immune cells between the two sample groups (**Figure 8B**). Compared with the normal samples, the IDD samples showed increased infiltration of CD8^+^T cells and M0 macrophages, whereas CD4 memory resting T cells, neutrophils, resting dendritic cells, follicular helper T cells, and monocytes showed decreased infiltration (**Figure 8C**).

Finally, the heat map revealed the correlation between the hub genes and immune cell infiltration (**Figure 8D**). As shown, the expression of hub genes was significantly correlated with immune cell infiltration across the subtypes. The reduced CD4 memory resting T cells in IDD samples are negatively correlated with highly expressed *CAPN1* and *EGFR* and positively correlated with *MAPK8*, *CTSB*, and *PRKCD* expression.

### Potential mRNA–miRNA–lncRNA (ceRNA) network of hub genes

**Figure 9:**
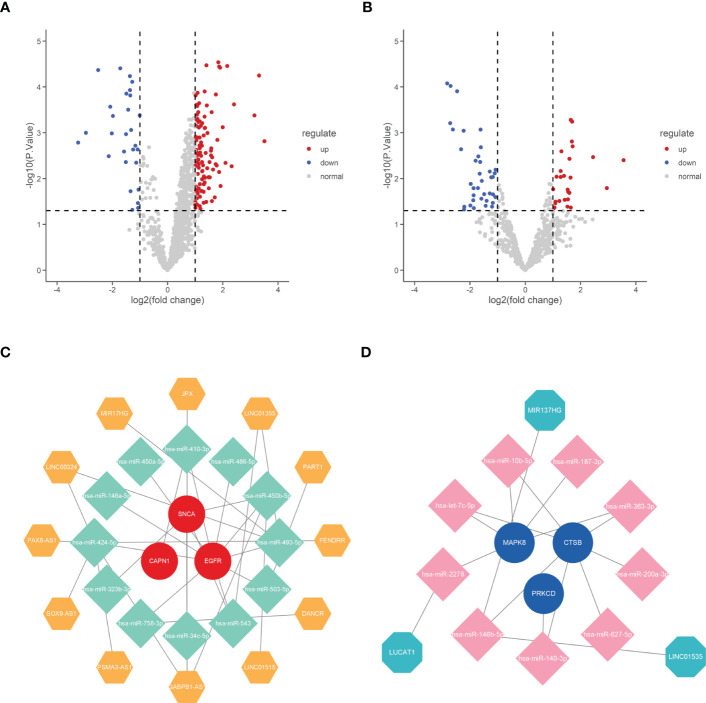
Construction of ceRNA network of the six hub genes. **(A)** Volcano plot of differentially expressed lncRNAs between normal and IDD samples. **(B)** Volcano plot of differentially expressed miRNAs between normal and IDD samples. **(C)** The ceRNA network of upregulated expression of hub genes. **(D)** The ceRNA network of downregulated expression of hub genes. IDD, intervertebral disc degeneration; ceRNA, competing endogenous RNA; lncRNA, long non-coding RNA; miRNA, microRNA.

To reveal the potential post-transcriptional regulatory mechanisms of these six hub genes, we screened differentially expressed miRNAs and lncRNAs during IDD development and constructed a ceRNA network. DE-miRNAs and DE-lncRNAs of GSE167199 were identified and screened in R software using the limma package, with |log2FC| > 1 and *p* < 0.05 set as significance thresholds. A total of 139 DE-lncRNAs (**Supplementary Table S4**) and 65 DE-miRNAs (**Supplementary Table S5**) were identified (**Figures 9A, B**).

Subsequently, the six hub genes targeted miRNAs were predicted using the ENCORI online database and intersected with DE-miRNAs to obtain mRNA–miRNA binding pairs (**Supplementary Table S6**). Moreover, the mirNet database was used to predict the possible binding lncRNAs of DE-miRNAs screened in the previous step, and the predicted lncRNAs were intersected with DE-lncRNAs to construct miRNA–lncRNA binding pairs (**Supplementary Table S7**). The mRNA–miRNA and miRNA–lncRNA binding pairs were then integrated to construct the ceRNA network of the six hub genes (**Figures 9C, D**).

### Validation of hub genes

**Figure 10:**
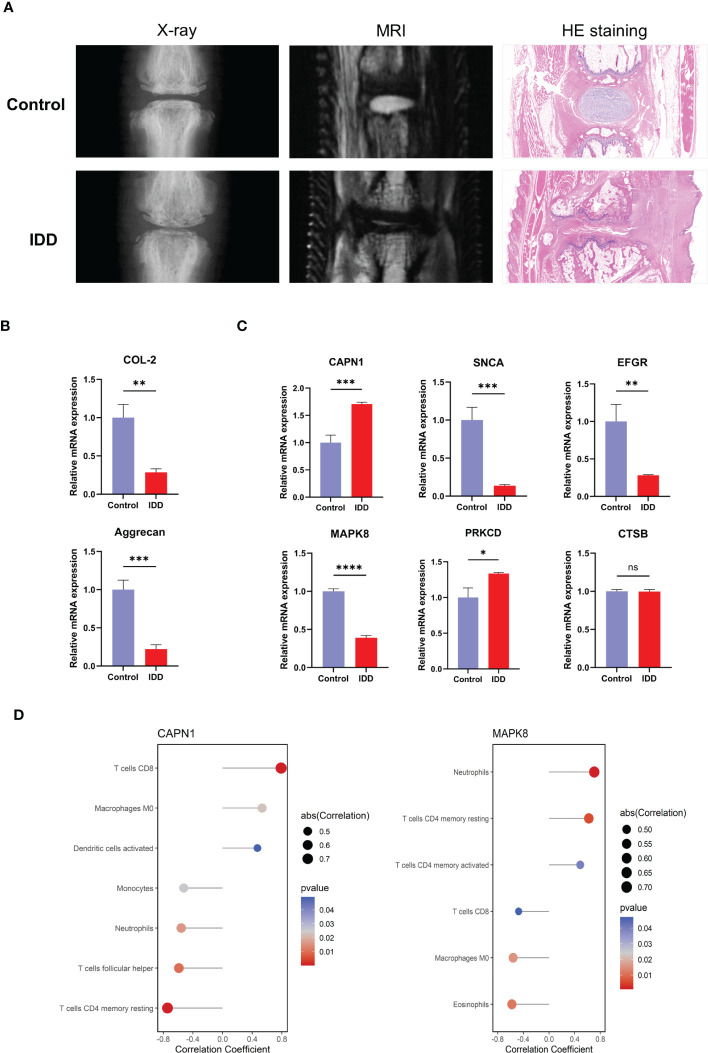
Expression levels of the hub genes in normal rat and IDD models. **(A)** Representative images of X-ray, MRI and H&E staining after needle puncture **(B)** The mRNA expression level of marker genes of NP. **(C)** The mRNA expression of the six hub genes in normal and IDD models. **(D)** CAPN1 and MAPK8 were analyzed for correlation with immune infiltration, and the analysis results of p < 0.05 were retained. H&E, hematoxylin and eosin; IDD, intervertebral disc degeneration; mRNA, messenger RNA. (* p < 0.05, ** p < 0.01, *** p < 0.001, **** p < 0.0001; ns, not significant).

According to the results, X-ray, MRI, and H&E staining of NP tissue sections revealed a severely damaged NP in IDD rat models (**Figure 10A**). Reverse transcription qPCR (RT-qPCR) analysis showed that the mRNA levels of aggregated proteoglycan (aggrecan) and type II collagen (COL-2) were decreased in IDD models (**Figure 10B**). We examined the mRNA expression of the hub genes in the IDD model. As the results showed, unlike other hub genes, only the expressions of *CAPN1* and *MAPK8* were consistent with our DEGs analysis results (**Figure 10C**). In addition, we also performed correlation analysis of *CAPN1* and *MAPK8* with immune cell subtype infiltration using *p* < 0.05 as screening threshold (**Figure 10D**). Therefore, *CAPN1* and *MAPK8* were validated as the final hub genes in the progression of IDD.

## Discussion

LBP is one of the most common health problems and represents a significant economic and lifestyle burden to human society, affecting approximately 60%–80% of the global population (28–30). IDD is one of the most important factors in the pathogenesis of LBP, and the application of high-throughput sequencing technology combined with bioinformatics analysis could help us to identify the biomarkers or key biological functions during IDD development and provide new ideas and options for IDD treatment (31, 32).

Autophagy, a highly conserved self-phagocytosis process in eukaryotic cells, prevents excessive apoptosis and promotes the secretion of the ECM in NP cells, and therefore plays a protective role in disc degeneration (20). Several compounds, such as autophagy agonists, can attenuate IDD by promoting autophagy to reduce oxidative stress, apoptosis, and ECM degradation in NP cells (33).

The NP tissue of the disc is surrounded by annulus fibrosus and cartilage endplate, a unique structure that sets NP cells and ECM in this tissue apart from the immune cells. However, when degeneration occurs, the NP outflow is recognized as a “foreign antigen” when it comes into contact with the external immune system, and an autoimmune response is generated to initial immune response. Once the NP tissue is damaged, the granulation tissue is formed, allowing the external blood vessels to extend and causing the NP tissue to be exposed to external immune cells. At this point, the ECMs secreted by NP cells, such as collagen and proteoglycan, are recognized as autoantigens and trigger a secondary immune response mediated by cytotoxic T cells (34, 35). Therefore, identifying the molecules related to inflammatory environment and immune cell infiltration in degenerating discs is important for revealing the underlying mechanisms of IDD pathogenesis.

In this study, we performed KEGG and GO enrichment analyses with the 636 obtained DEGs from the IDD database. The KEGG results showed that many immune-related disease pathways were highly activated in the IDD group, such as shigellosis, *Salmonella* infection, and Yersinia infection. Previous studies have shown that bacterial infections, such as *Staphylococcus aureus* and *Cutibacterium acnes*, create an inflammatory environment in the intervertebral disc and promote IDD progression (36–38).

Moreover, GO results demonstrate that autophagy plays a central role during IDD development. Thus, based on above results, we next screened for only autophagy-related genes in DEGs and explored the possible core DEGs. Using the MCODE plug-in of Cytoscape software, we successfully screened the core gene clusters of 30 DE-ARGs. Combined with PPI network identification, we selected six genes as the hub genes, that is *MAPK8*, *CTSB*, *PRKCD*, *SNCA*, *CAPN1*, and *EGFR*. The expression of these six hub genes in the original raw data was significantly different between the control and IDD samples.

CIBERSORT is an inverse convolution analysis algorithm based on linear support vector regression that estimates the relative abundance of immune cells in a mixed cell population by analyzing gene expression data (39). Using this algorithm, we found that there was increased infiltration of CD8^+^ T cells and M0 macrophages in IDD samples, and decreased infiltration of CD4^+^ memory T cells, neutrophils, resting dendritic cells, follicular helper T cells, and monocytes. Correlation analysis of the expression of six hub genes with the relative abundance of 22 immune cell types revealed that the hub genes were significantly correlated with several immune cell types that were increased or decreased in the IDD group. The increased infiltration of CD4^+^ and CD8^+^ T cells has been reported in spontaneous disc herniation in the human tumor necrosis factor alpha (TNF-α)-overexpressing transgenic mouse model (Tg197) (40). Thus, we speculate that the efflux of NP from the degenerating disc and exposure to the external immune environment generates an inflammatory environment. Released pro-inflammatory cytokines promote the infiltration of other immune cells, regulate hub gene expression and the biological behavior of NP cells, maintain the inflammatory microenvironment, and ultimately exacerbate disc herniation.

Non-coding RNAs, such as lncRNAs and miRNAs, have also attracted widespread attention for their role in mediating autophagy in IDD (41–43). Non-coding RNAs can regulate the degree of activation of autophagy in immune cells by directly or indirectly targeting autophagy-related genes and the associated signaling pathways. However, relatively little is known about the co-regulation mechanisms of non-coding RNAs. LncRNAs, microRNAs, and mRNAs can regulate the development and progression of IDD by forming ceRNA networks (21, 44, 45). The GSE167199 dataset used in this study was also used previously for the study of ceRNA networks and identified two ceRNA axes, lncRNA XIST-hsa-miR-4775-PLA2G7 and lncRNA XIST-hsa-miR-424–5p-AMOT/TGFBR3, that may be involved in the progression of IDD (46). However, the experimental results were mainly based on one RNA sequencing dataset. They did not involve online databases for target prediction, so multiple samples and online databases need to be combined for comprehensive analysis. The ceRNA networks regulating IDD’s molecular mechanisms remain to be further investigated and explored.

Through differential analysis of gene expression profiles downloaded from the GEO database and online database prediction, we identified several pairs of ceRNA axis and constructed ceRNA regulatory networks for the six hub genes. Among the ceRNA networks, several miRNAs and lncRNAs have been reported in the literature to be involved in disease regulation. In NP cells, the overexpression of LINC00324 increases Fas ligand (FasL) expression and promotes disc degeneration (47). The miR-140–3p affects bone marrow stromal cells (BMSCs) in degenerative intervertebral disc disease (IVD) by directly targeting KLF5 and interacting with the N–cadherin/MDM2/Slug axis, thereby regulating regenerative effects in degenerative IVD (48). LINC01535 affects clear cell renal cell carcinoma progression by mediating PI3K/Akt signaling through the LINC01535/miR–146b-5p/TRIM2 axis (49), corresponding to the LINC01535/miR-146b-5p binding pair predicted in this study. Although there are no studies on the relationship between LINC01535 and IDD, based on the results of previous studies and bioinformatic analysis, it is reasonable to believe that the ceRNA axis, composed of LINC01535 and several other non-coding RNAs, is involved in the regulatory network on disc degeneration.

Finally, we designed *in vitro* experiments to verify whether or not the hub genes were differentially expressed in the NP of the IDD rat model. Based on the RT-qPCR results, we found that two hub genes, *MAPK8* and *CAPN1*, were differentially expressed in the IDD and control groups, and that these expression trends were consistent with the results of previous bioinformatics analysis.

*MAPK8*, also known as c-JUN N-terminal kinase (*JNK*), is a member of the MAPK family that regulates a variety of physiological responses, including inflammatory responses, cell differentiation, cell proliferation, and cell death. The dysregulation of MAPK8 has also been implicated in several diseases, including diabetes, cancer, autoimmune diseases, cardiac hypertrophy, and asthma (50). Previous studies have shown that *MAPK8* also has some relevance to the physiological state of NP cells. In human NP cells, the use of JNK pathway inhibitors can counteract interleukin 17 (IL-17)-induced COX2/PGE2 production and IVD inflammation, which may be a potential therapeutic target for alleviating IDD (51). At high osmotic pressure, the *JNK* pathway can regulate cell generation, proliferation, and apoptosis in rabbit NP cells, which helps to elucidate the pathological mechanisms involved in intervertebral discs under elevated osmotic pressure and load (52). In another study, the development of disc degeneration in rabbits was prevented by regulating the JNK signaling pathway and the downstream p53 pathway, in which *JNK*/*p53* plays an important role (53). Therefore, *MAPK8* could be further investigated as a potential biomarker for IDD.

Calpain 1 (*CAPN1*), an intracellular cysteine protease, is ubiquitously expressed in mammals, and is involved in the cleavage of cytoskeletal, mitochondrial, and lysosomal membrane proteins and mediated impairment of autophagic flux to neurons (54). Virus-infected cardiomyocytes induce inflammation in an immune-mediated manner *via NLRP3* inflammasome. *CAPN1* inhibitors can inhibit *NLRP3* inflammasome release and alleviate myocardial injury (55). *CAPN1* has been well studied in autophagy-related diseases, but whether or not it impacts the progress of IDD, or whether the signal pathway related to *CAPN1* regulates autophagy activity and finally acts on the degenerated NP, has not been reported on in detail in published studies and needs further research.

Of course, this study inevitably has some limitations. First, the sample size of gene expression profiles downloaded from public databases is slightly inadequate, and individual differences in the samples may affect the analysis results’ generalizability. In addition, only the mRNA levels and not the protein levels of the hub gene were validated by RT-qPCR. More relevant *in vivo* and *in vitro* experiments are needed to demonstrate the role of these hub genes and their potential mechanisms in IDD. Finally, some of the lncRNAs and miRNAs obtained from the analysis have not been reported in IDD-related studies, and further experiments are needed for validation.

In summary, we analyzed the DEGs of IDD using bioinformatics methods, including the functional enrichment analysis of DEGs, acquisition of autophagy-related DEGs, PPI network analysis, and in vitro qPCR validation. In addition, we performed immune cell infiltration analysis and lncRNA–miRNA–mRNA network construction, and investigated the correlation between immune cell infiltration and ceRNA network in IDD. In animal experiments, we validated the expression of two hub genes (*MAPK8* and *CAPN1*) at the mRNA level, which was consistent with the results of bioinformatics analysis. Our study provides new insights into the pathogenesis of IDD and helps to identify new potential therapeutic targets in the pathogenesis of IDD.

## Data availability statement

The datasets presented in this study can be found in online repositories. The names of the repository/repositories and accession number(s) can be found in the article/**Supplementary Material**.

## Ethics statement

The animal study was reviewed and approved by Vital River Laboratory Animal Care and Use Committee.

## Author contributions

YXZ, JZ, and ZS contributed to the design of the experiments, analysis of the data, and writing of the article. HW and RN contributed to the validation of the experimental results. LX and YCZ contributed to the collection of the experimental data. KY, XX, and JT supervised the entire experimental process and helped revise the manuscript. All authors contributed to the article and approved the submitted version.
