# Supplementary material for: MAPK8 and CAPN1 as potential biomarkers of intervertebral disc degeneration overlapping immune infiltration, autophagy, and ceRNA
Source: Front Immunol. 2023 May 30;14:1188774. doi: 10.3389/fimmu.2023.1188774 (PMC10266224; doi:10.3389/fimmu.2023.1188774)
Supplement: Supplementary file 1 [file Table_1.docx]

Supplementary Material

**Supplementary Table S1.** The details of the GEO datasets used to analysis.

| **GEO Dataset** | **Platform** | **Type of tissue** | **IDD** | **Control** |
| --- | --- | --- | --- | --- |
| GSE176205 | Illumina HiSeq 4000 | Nucleus Pulposus | 6 | 3 |
| GSE167931 | HiSeq X Ten | Nucleus Pulposus | 5 | 4 |
| GSE167199 | Illumina NovaSeq 6000 | Nucleus Pulposus | 3 | 3 |
