# Supplementary material for: MAPK8 and CAPN1 as potential biomarkers of intervertebral disc degeneration overlapping immune infiltration, autophagy, and ceRNA
Source: Front Immunol. 2023 May 30;14:1188774. doi: 10.3389/fimmu.2023.1188774 (PMC10266224; doi:10.3389/fimmu.2023.1188774)
Supplement: Supplementary file 2 [file Table_2.docx]

Supplementary Material

**Supplementary Table S2.** Primers of genes.

| **Gene** | **Sequence** |
| --- | --- |
| Mapk8 | F : TTACTGTGTCACGCCATGCT |
|  | R : GAGCTTCTCTGTACTGGCGG |
| Ctsb | F : ATTCACACCAATGGCCGAGT |
|  | R : AGCCACCATTACAGCCATCC |
| Prkcd | F : TGCCTCACCGATTCAAGGTC |
|  | R : CCCTGTTTCACCAATCCCCA |
| Snca | F : ATGTCGTTGTACCCACTGTCC |
|  | R : ACTGTAGTGAGAGGGGAGCA |
| Capn1 | F : GCTCATCATCACCCGCTACTC |
|  | R : TCAAAGGTCACAACACCATCCA |
| Egfr | F : GGCATCATGGGGGAGAACAA |
|  | R : TCTTTGGCCCATAGGTACAGTT |
| Col2a1 | F : GCCAGGATGCCCGAAAATTAG |
|  | R : CTTGTCACCACGGTCACCTC |
| Acan | F :GGGACCTGTGTGAGATCGAC |
|  | R :GGTCGGGAAAGTGGCGATAA |
| Gapdh | F : TCTCTGCTCCTCCCTGTTCT |
|  | R : GTTCACACCGACCTTCACCA |
